# *In vitro* propagation, shoot regeneration, callus induction, and suspension from lamina explants of *Sorbus caloneura*

**DOI:** 10.48130/FR-2023-0007

**Published:** 2023-03-30

**Authors:** Miaomiao Guo, Qiuying Yu, Daijun Li, Kexin Xu, Zexin Di, Yong Zhang, Yang Yu, Jian Zheng, Yan Zhang

**Affiliations:** 1 School of Architecture, Beijing University of Agriculture, Beijing 102206, China; 2 Beijing Ming Tombs Forest Farm Management Office, Beijing 102200, China

**Keywords:** *Sorbus caloneura*, Callus, Proliferation, Regeneration, Suspension

## Abstract

*Sorbus caloneura* has high ornamental and medicinal value but is endangered, and significant effort is required to preserve this natural resource. In this study, the stem and sterilized leaves of *S*.* caloneura* were used to explore the effects of different plant hormones basic medium type, initial callus quality, initial liquid volume, and ratio of old liquid culture medium to new on stem proliferation, regeneration and callus suspension culture. Naphthylacetic acid (NAA) and 6-benzyladenine (6-BA) significantly affected the proliferation of stem tissue. Murashige and Skoog (MS) basal medium supplemented with 1.75 mg/L 6-BA, 0.25 mg/L NAA, and 0.25 mg/L indole butyric acid (IBA) yielded a proliferation rate of 100%, with an average number of adventitious shoots per stem of 4.9. The best callus induction was observed with MS basal medium containing 0.5 mg/L 6-BA, 1 mg/L 2,4-dichlorophenoxyacetic acid (2,4-D), and 0.2 mg/L thidiazuron (TDZ). The adventitious shoots were directly induced by MS basal medium containing 5.0 mg/L 6-BA, 0.5 mg/L NAA, and 1.5 mg/L kinetin (KT) and indirectly induced by medium containing 3.0 mg/L 6-BA, 0.3 mg/L NAA, and 0.1 mg/L TDZ. NAA significantly affected rooting rate, with ideal conditions found to be medium supplemented with 0.2 mg/L NAA and 1.5 mg/L IBA. MS basal medium with 4.5 g of calli and 120 mL of liquid culture medium without retention of the original culture medium yielded the best suspension effect for callus proliferation. Taken together, the results of this study lay a foundation for the breeding, preservation of germplasm resources, and genetic transformation of* S*.* caloneura.*

## INTRODUCTION

*Sorbus* is a genus of deciduous shrubs and small trees belonging to the Rosaceae family that are valued for their ornamental, ecological, and medicinal properties. Plants of this genus are popular worldwide, especially in central European countries, and are used in traditional Chinese and North American medicine^[1,2]^. *Sorbus* *caloneura* is an ornamental tree of the *Micromeles* section^[3]^, which is distributed across southwest China and northern Vietnam and is commonly found in river valleys and mountains. It is also an endangered species in Fujian Province, China; thus, it is essential to preserve the existing germplasm and increase the population of this highly valuable tree species.

*In vitro* plant regeneration has been shown to be critical for large-scale commercial production, germplasm preservation, and plant improvement^[4]^. The efficiency of shoot regeneration of woody plants is influenced by the type of explant, composition of the basal medium, and admixture of phytohormones and growth additives^[5−7]^. Due to the long growth cycle of woody plants, *in vitro* propagation techniques are limited by a lack of understanding of growth rhythms, dormancy, and production of phenolic compounds^[8]^.

*In vitro* shoot regeneration has only been successfully achieved in a few *Sorbus* species such as *S*.* pohuashanensis*, *S*. *aucuparia*, *S*. *folgneri*, and *S*. *alnifolia*^[9−13]^. In addition, leaves, petioles, and cotyledons have been used as explants to induce adventitious shoots in* Sorbus*^[10,11,13]^. Moreover, the type and concentration of cytokinin and auxin used during shoot regeneration have been shown to significantly impact its success rate^[13,14]^. However, at present, there are no reports of shoot regeneration in *S.* *caloneura*.

Plant cell suspension culture is an efficient technology that has been widely used in protoplast isolation, culture and hybridization, gene transfer, and rapid production of secondary metabolites^[15−17]^. Many suspension culture systems have been established for woody plants such as *Houpoea officinalis*^[18]^, *Eucommia ulmoides*^[19]^, and *Populus tomentosa*^[20]^. The main factors affecting the suspension culture of woody plants are plant material, medium composition, initial inoculum, shaking speed, length of subculture period, and ratio of old-to-new medium^[21−24]^. In *Sorbus*, suspension cultures of *S. aucuparia* and *S. pohuashanensis* were established to explore different types of inducers that stimulate plant suspension cells to initiate different pathways to produce and accumulate secondary metabolites with antibacterial activity such as biphenyls and dibenzofurans. These compounds are utilized by plants as chemical defence measures to counteract both abiotic and biotic stresses^[25−27]^. However, suspension culture technology has not yet been applied to *S. caloneura*.

In this study, stem segments and sterile leaves of *S. caloneura* were used as explants to explore the effects of plant hormones and other conditions on stem segment proliferation, callus induction, suspension culture, and shoot regeneration. The resulting protocol will be useful for *Sorbus* breeding and genetic modification.

## MATERIALS AND METHODS

### Plant materials and explant manipulation

**Figure 1:**
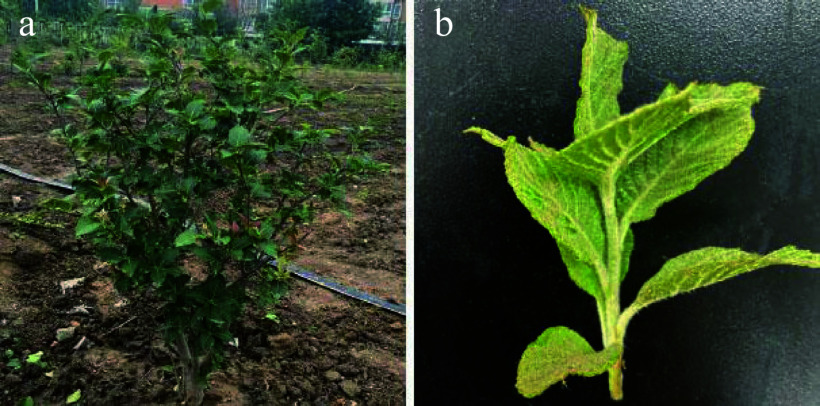
Morphology of young stems and leaves of* Sorbus caloneura.* (a) Four-year-old *S. caloneura* plant. (b) Young stems and leaves.

In this study, *Sorbus caloneura* stems were collected from four-year-old plants with a height of 90–120 cm that were cultivated at Beijing University of Agriculture (40° N, 116° E, Beijing, China) from May to June 2021 (Fig. 1).

The collected stems were cut into small segments of approximately 2–3 cm, cleaned with detergent, soaked for 10 min in detergent solution to remove surface material, and subsequently washed under running water for 1–1.5 h. After treatment, the stem segments were transferred to a clean bench for disinfection. The disinfection procedure was as follows: stem segments with shoots were soaked in 75% alcohol for 1 min, sterilized with 0.2% w/v sodium hypochlorite solution for 12 min, washed three times with sterile distilled water, and inoculated into the proliferation medium that was composed of MS basal medium^[28]^ supplemented with 1.25 mg/L 6-benzyladenine (6-BA), 0.25 mg/L naphthylacetic acid (NAA), 0.25 mg/L indole butyric acid (IBA), and 30 g/L sucrose + 6.5 g/L agar. All media in this study contained the same sucrose and agar content. The cultures were incubated at 25 ± 2 °C with a 16-h photoperiod under fluorescent light at 20 μmol/m^2^/s.

### Stem proliferation

Stems were subcultured every four weeks and cultured on stem proliferation medium (MS basal medium supplemented with 1.25 mg/L 6-BA, 0.25 mg/L NAA, and 0.25 mg/L IBA). Sterile stem segments (≥ 2 cm) from the subculture were used as explants that were transferred to MS basal medium supplemented with 0.25 mg/L IBA, 6-BA (1.25 or 1.75 mg/L), NAA (0.25 or 0.50 mg/L), and 30 g/L sucrose + 6.50 g/L agar (Table 1). The pH of all media was adjusted to 5.8. Each of the four combinations of growth regulators were tested in triplicate, with each test containing 10 explants. The cultures were then incubated at 25 ± 2 °C with a 16-h photoperiod for 30 d. The number of adventitious shoots (shoot height ≥ 1 cm) were counted, and the rates of proliferation and vitrification were calculated using the following formulae:

Proliferation rate (%) = (number of proliferation shoots / number of primary explants) × 100%

Vitrification (%) = (number of vitrificated explants / induced explants) × 100%

**Table 1:** Effects of 6-BA and NAA on stem proliferation.

Medium	6-BA (mg/L)	NAA (mg/L)	Number of shoots/ explant	Proliferation rate (%)	Vitrification (%)
1	1.25	0.25	2.77 ± 0.31c	100	0.00 ± 0.00b
2	1.25	0.50	3.30 ± 0.46bc	100	0.00 ± 0.00b
3	1.75	0.25	4.90 ± 0.10a	100	13.33 ± 5.77a
4	1.75	0.50	3.37 ± 0.15b	100	20.00 ± 10.00a
Data are represented as the mean ± SE of three replicates. Different lowercase letters indicate significant differences among treatments as determined by Duncan's test (*P* ≤ 0.05). Abbreviations: SE = standard error; 6-BA = 6-benzyladenine; NAA = naphthylacetic acid.					

### Direct shoot regeneration

**Figure 2:**
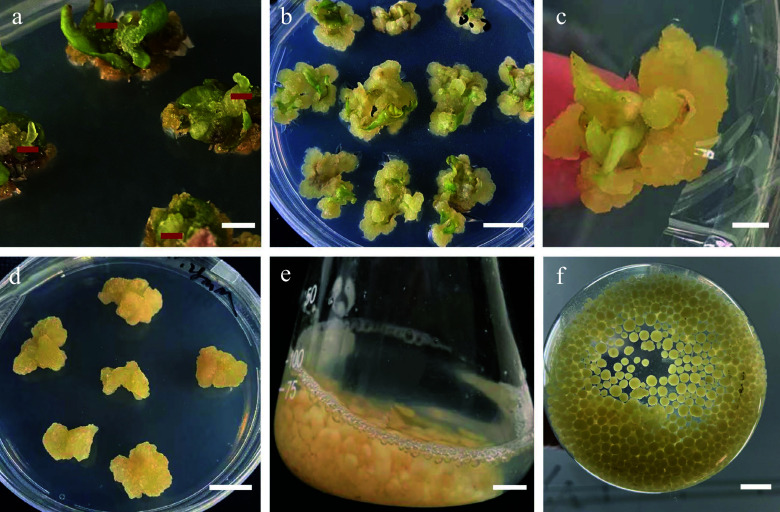
Morphology of callus induction, shoot regeneration, and callus suspension. (a) Morphology of the leaf samples that were proximal halves cut twice vertically through the main vein. (b) Direct adventitious shoot regeneration (red mark). (c) Calli induced from leaves. (d) Callus adventitious shoot regeneration from callus. (e) Calli proliferation in suspension culture. (f) Suspension subculture. Scale bars (a), (b), (d) = 0.5 cm, (c), (e), (f) = 1 cm.

The leaf samples were proximal halves cut twice vertically through the main vein (Fig. 2a). These samples were placed in shoot regeneration medium, with the adaxial side in contact with the medium.

Different concentrations of 6-BA (1.00, 3.00, or 5.00 mg/L), NAA (0.10, 0.25, or 0.50 mg/L), and kinetin (KT) (1.00, 1.50, or 2.00 mg/L) were added to the medium in combination to determine the optimum combination of growth regulators for a total of nine treatments (Table 2). Each combination was tested in triplicate, with each test containing 10 explants, and the adventitious shoot regeneration rate was calculated using the following formula:

Adventitious shoot regeneration rate (%) = (number of explants with adventitious shoots / total number of explants) × 100%

**Table 2:** Effects of 6-BA, NAA, and KT on direct shoot regeneration from leaves.

Medium	6-BA (mg/L)	NAA (mg/L)	KT (mg/L)	Regeneration rate (%)	Growth status of explants
1	1.00	0.10	1.00	0.00 ± 0.00b	Poor and dense horizontal distribution of yellowish-brown callus
2	1.00	0.25	1.50	0.00 ± 0.00b	General, yellow-green dense callus distributed in a few spots
3	1.00	0.50	2.00	0.00 ± 0.00b	General, yellow-green dense callus
4	3.00	0.10	1.50	0.00 ± 0.00b	General, light-yellow dense callus
5	3.00	0.25	2.00	0.00 ± 0.00b	Poor, yellow-brown callus, denser
6	3.00	0.50	1.00	0.00 ± 0.00b	General, light-yellow dense callus
7	5.00	0.10	2.00	0.00 ± 0.00b	Grew well, callus was loose when it was yellowish and healed
8	5.00	0.25	1.00	0.00 ± 0.00b	Good growth, shoot point appeared
9	5.00	0.50	1.50	20.00 ± 0.20a	Grew well, adventitious shoots detected
Data are represented as the mean ± SE of three replicates. Different lowercase letters indicate significant differences among treatments as determined by Duncan’s test (*P* ≤ 0.05). Abbreviations: SE = standard error; 6-BA = 6-benzyladenine; NAA = naphthylacetic acid; KT = kinetin.					

### Callus induction

Sterile leaves from the adventitious shoots that were subcultured on new medium for 15 d were used as explants. The main veins were cut 1–2 times, and the leaves were placed in medium with the paraxial surface facing up. The medium was composed of MS basal medium supplemented with 6-BA (0.10, 0.50, or 2.00 mg/L), 2,4-D (0.10, 1.00, or 2.00 mg/L), and TDZ (0.10, 0.20, or 0.50 mg/L). The test was conducted using a randomized block design for a total of 27 treatments (Table 3). Each combination was tested in triplicate, with each test containing 10 explants. Callus growth was observed and recorded after 20 d. The callus induction rate was calculated using the following formula:

Callus induction rate (%) = (number of inducted callus explants / total number of explants) × 100%

**Table 3:** Effects of 6-BA, 2, 4-D, and TDZ on leaf callus induction.

Medium	6-BA (mg/L)	2,4-D (mg/L)	TDZ (mg/L)	Induction rate (%)	Growth status of explant
1	0.10	0.10	0.10	23.33 ± 3.83e	Poor growth, little yellowish-brown calli with uneven distribution
2	0.10	0.10	0.20	83.33 ± 4.70c	General growth, swelling, fewer light-yellow calli
3	0.10	0.10	0.50	96.67 ± 10.64ab	General growth, yellowish and whitish relatively compact calli, with small white particles in bulges
4	0.10	1.00	0.10	90.00 ± 13.61bc	Poor growth, almost no calli
5	0.10	1.00	0.20	96.67 ± 10.64ab	General growth, yellow compact calli with uniform distribution
6	0.10	1.00	0.50	90.00 ± 13.61bc	General growth, yellowish compact calli and brown leaves without calli
7	0.10	2.00	0.10	100.00 ± 0.00a	General growth, obvious wounds, light-green with yellow, more compact calli
8	0.10	2.00	0.20	100.00 ± 0.00a	General growth, many pale-yellow dense calli over the leaves
9	0.10	2.00	0.50	86.67 ± 4.70c	Good growth, yellow-green, rapid proliferation and vitrification
10	0.50	0.10	0.10	100.00 ± 0.00a	General growth, yellow-green, relatively compact calli, many bulges
11	0.50	0.10	0.20	100.00 ± 0.00a	Good growth, yellow-green, compact, evenly covered with calli at the incision
12	0.50	0.10	0.50	100.00 ± 0.00a	Good growth, loose and yellowish-green granular calli with even distribution
13	0.50	1.00	0.10	100.00 ± 0.00a	General growth
14	0.50	1.00	0.20	100.00 ± 0.00a	Good growth, loose yellowish calli
15	0.50	1.00	0.50	100.00 ± 0.00a	Good growth, loose yellow-green granular calli
16	0.50	2.00	0.10	100.00 ± 0.00a	Good growth, full of calli, granular, loose, yellow-green, with white base
17	0.50	2.00	0.20	100.00 ± 0.00a	General growth, yellowish compact calli
18	0.50	2.00	0.50	100.00 ± 0.00a	Weak growth, yellowish and whitish with even distribution, tended to turn brown
19	2.00	0.10	0.10	86.67 ± 4.70c	General growth, few scattered yellow-green calli
20	2.00	0.10	0.20	100.00 ± 0.00a	Poor growth, banded or small calli
21	2.00	0.10	0.50	63.33 ± 3.48d	General growth, light-yellow loose calli only appeared at the incision, and calli turned brown gradually
22	2.00	1.00	0.10	100.00 ± 0.00a	Poor growth, slightly whiter yellow band, slower emergence, browning of leaf edge, denser and smaller particles
23	2.00	1.00	0.20	100.00 ± 0.00a	Poor growth, pale yellow with white, waterlogged, small patches
24	2.00	1.00	0.50	96.67 ± 10.64ab	Poor growth, dense cluster distribution, and little browning
25	2.00	2.00	0.10	100.00 ± 0.00a	Poor growth, light green and more brown granular calli, severe browning of leaves, slower emergence
26	2.00	2.00	0.20	96.67 ± 10.64ab	General growth, yellow-green brownish and compact calli
27	2.00	2.00	0.50	96.67 ± 10.64ab	General growth, large leaf curl, uniform distribution of yellow-brown calli
Data are represented as the mean ± SE of three replicates. Different lowercase letters indicate significant differences among treatments as determined by Duncan’s test (*P* ≤ 0.05). Abbreviations: SE = standard error; 6-BA = 6-benzyladenine; NAA = naphthylacetic acid; KT = kinetin.					

### Indirect shoot regeneration

Different concentrations of 6-BA (1.00, 3.00, or 5.00 mg/L), NAA (0.10, 0.30, or 0.50 mg/L), and TDZ (0.10 mg/L) were added to the medium in combination to determine the optimum combination of growth regulators for a total of nine treatments (Table 4). After subculturing in callus induction medium for 20 d, the calli of the group with the best callus induction were transferred to the regeneration medium. After 40–45 d, leaf differentiation was observed, and the number of adventitious shoots (shoot length > 1 cm) were counted. The adventitious shoot regeneration rate was calculated as described above.

**Table 4:** Effects of 6-BA and NAA on indirect shoot regeneration from calli.

Medium	6-BA (mg/L)	NAA (mg/L)	Regeneration rate (%)
1	1.00	0.10	0.00 ± 0.00a
2	1.00	0.30	0.00 ± 0.00a
3	1.00	0.50	0.00 ± 0.00a
4	3.00	0.10	0.00 ± 0.00a
5	3.00	0.30	3.33 ± 0.19a
6	3.00	0.50	0.00 ± 0.00a
7	5.00	0.10	0.00 ± 0.00a
8	5.00	0.30	0.00 ± 0.00a
9	5.00	0.50	0.00 ± 0.00a
Data are represented as the mean ± SE of three replicates. Different lowercase letters indicate significant differences among treatments as determined by Duncan’s test (*P* ≤ 0.05). Abbreviations: SE = standard error; 6-BA = 6-benzyladenine; NAA = naphthylacetic acid.			

### Rooting culture

Healthy stem segments (> 2 cm) were inoculated into 1/2 MS rooting medium containing auxin, NAA (0, 0.20, 0.40, or 0.80 mg/L), and IBA (1.00 or 1.50 mg/L) for a total of eight treatments (Table 5). The rooting morphology was observed, and the rooting number was recorded after 25 d. The rooting rate was calculated using the following formula:

Rooting rate (%) = (number of rooting plantlets / all shoots) × 100%

**Table 5:** Effects of NAA and IBA on rooting of *S.caloneura.*

	NAA (mg/L)	IBA (mg/L)	Rooting Rate (%)	Rooting number (strip)	Rooting status
1	0.00	1.00	26.67 ± 2.29cd	2.50 ± 0.13cd	Light-green with white roots, thick, and more extensive aboveground parts
2	0.00	1.50	36.67 ± 3.89c	2.75 ± 0.09c	Light-green with white roots, thick, and more extensive aboveground parts
3	0.20	1.00	56.67 ± 2.03b	4.11 ± 0.06b	White main root, many lateral roots
4	0.20	1.50	76.67 ± 4.43a	4.78 ± 0.12a	White main root, many lateral roots
5	0.40	1.00	23.33 ± 3.83de	2.29 ± 0.15d	White main root, stout, with lateral roots
6	0.40	1.50	6.67 ± 8.66f	1.50 ± 0.50ef	White stout short root
7	0.80	1.00	16.67 ± 2.74de	1.20 ± 0.20f	White main root, few lateral roots
8	0.80	1.50	13.33 ± 2.96e	1.75 ± 0.25e	White roots with browning at the base
Data are represented as the mean ± SE of three replicates. Different lowercase letters indicate significant differences among treatments as determined by Duncan's test (*P* ≤ 0.05). Abbreviations: SE = standard error; NAA = naphthylacetic acid; IBA = indole butyric acid.					

### Suspension culture

A light-yellow callus of *S*. *caloneura* with a soft texture and vigorous growth (less than three subcultures) was selected as the material for suspension culture, with an orthogonal experimental design L_9_(3^4^) (Table 6). Several parameters were assessed, including basic medium type (A), initial callus quality (B), initial liquid volume (C), and ratio of old-to-new liquid culture medium (D). Each treatment was repeated three times. The suspension was subcultured for 15 d in a 200 mL volume flask at a rotating speed of 120 rpm, and the amount of callus proliferation was calculated. The basic medium was composed of MS basal medium supplemented with 0.50 mg/L 2,4-D, and 0.05 mg/L TDZ. In a previous preliminary experiment, we found that the weight of calli tended to stabilize after approximately 15 d of culture; therefore, we chose to measure the amount of callus proliferation at 15 d and 30 d after suspension culture.

**Table 6:** Influencing factors and level of orthogonal test for callus suspension.

Level	Factor			
	A	B	C	D
	Basic medium type	Initial callus quality (g)	Initial liquid volume (mL)	Ratio of old-to-new liquid culture medium
1	MS	1.5	120	0
2	WPM	3.0	100	1/3
3	½MS	4.5	80	1/1
Abbreviations: MS = Murashige and Skoog; WPM = woody plant medium; ½MS = ½Murashige and Skoog.				

### Data statistics and analysis

Analysis of variance (ANOVA) was performed using SPSS version 18.0 (SPSS, Chicago, IL, USA). Arcsine transformation was applied to the percentage data before ANOVA using Microsoft Excel 2013 (Microsoft Corp., Richmond, VA, USA). The data were then subjected to one-way ANOVA, followed by Duncan's multiple range test at *P* ≤ 0.05 and expressed as the mean ± standard error.

## RESULTS

### Effects of 6-BA and NAA on stem proliferation

**Figure 3:**
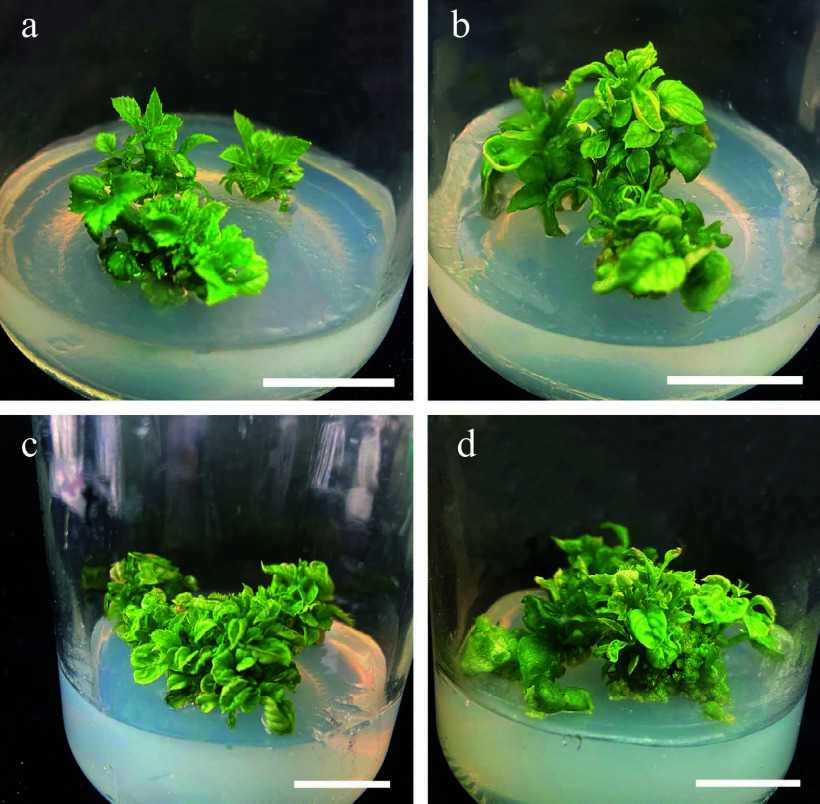
Effects of different concentrations of 6-BA and NAA on the proliferation of stems. (a) 6-BA (1.25 mg/L) and NAA (0.25 mg/L). (b) 6-BA (1.25 mg/L) and NAA (0.50 mg/L). (c) 6-BA (1.75 mg/L) and NAA (0.25 mg/L). (d) 6-BA (1.75 mg/L) and NAA (0.50 mg/L), Scale bars = 2 cm.

Samples grown on MS basal medium supplemented with 1.75 mg/L 6-BA, 0.25 mg/L NAA, 0.25 mg/L IBA had proliferation rates of 100% and an average of 4.9 adventitious shoots, which was significantly higher than that of the other three groups (Table 1). Visual inspection indicated that these explants had vigorous growth, with bright green leaves and stems (Fig. 3). ANOVA showed that 6-BA significantly affected the average number of shoots (Table 1). However, vitrification markedly increased as the concentration of 6-BA increased, reaching 20% at the highest 6-BA concentration (Table 7).

**Table 7:** Variation analyses of the average number of shoots for different 6-BA and NAA combinations.

Variation source	*df*	MS	F	*P*-value
6-BA	1	3.641	11.614	0.008*
NAA	1	0.603	1.932	0.199
Error	9	0.314		
Total	12			
* Represents a significant difference at *P* < 0.05. ** Represents a highly significant difference at *P* < 0.01. Abbreviations: *df* = degree of freedom; MS = mean square; 6-BA = 6-benzyladenine; NAA = naphthylacetic acid.				

### Effects of phytohormones on leaf regeneration

Our results demonstrated that hormone treatment had a positive effect on the number of adventitious buds induced directly from *S. caloneura* leaves. We employed three different concentrations of 6-BA, NAA, and KT in several different combinations, one of which resulted in direct adventitious shoots and a leaf regeneration rate of 20% (Fig. 3b, Table 2).

We found that increasing concentrations of 2,4-D during the callus induction of leaves resulted in a larger calli volume. After two weeks of growth, explants were inoculated into callus induction medium. In the third week, granular and compact expanded calli formed at the cut site of the explants (Fig. 3c). The best callus induction medium for leaves was MS basal medium supplemented with 0.50 mg/L 6-BA, 1.00 mg/L 2,4-D, and 0.20 mg/L TDZ, with a callus induction rate of 100% (Table 3). The induction rate increased gradually with decreasing cytokinin-to-auxin ratios. The calli of the explants grew well when the concentration of 6-BA was 0.50 mg/L, with yellowish-green colour, granular distribution, and loose structure.

We achieved indirect adventitious bud differentiation from the calli of *S. caloneura*. MS basal medium supplemented with 0.50 mg/L 6-BA, 1.00 mg/L 2,4-D, and 0.20 mg/L TDZ resulted in calli that differentiated into adventitious shoots with media containing 3.00 mg/L 6-BA, 0.30 mg/L NAA, and 0.1 mg/L TDZ, which was the only combination of growth regulators that resulted in differentiation into adventitious shoots (Fig. 3d). However, this combination resulted in a regeneration rate of only 3.33% (Table 4).

### Effects of NAA and IBA on rooting culture

**Figure 4:**
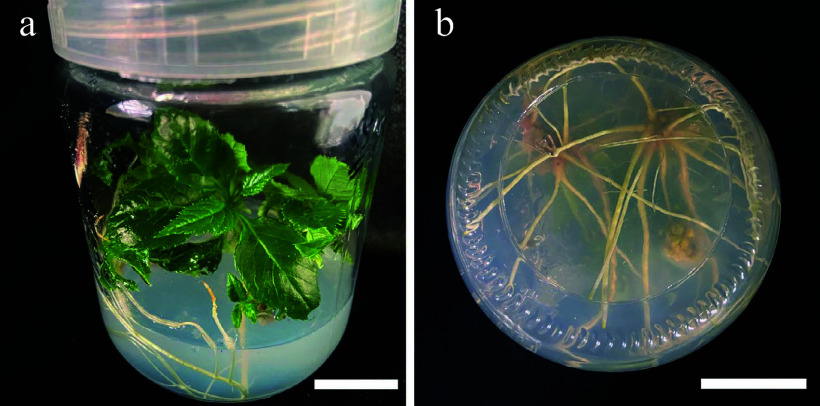
Morphology of roots treated with NAA (0.20 mg/L) and IBA (1.50 mg/L). (a) 30 d old root, front view. (b) 30 d old root, bottom view. Scale bars = 2 cm.

ANOVA analysis indicated that NAA had a significant effect on the rooting of *S. caloneura* (Table 8), but all eight media combinations tested resulted in some level of rooting (Table 5). The combination of 1/2 MS with 0.20 mg/L NAA and 1.50 mg/L IBA resulted in a rooting rate of 76.67% and a rooting number of 4.78, indicating that it was the most suitable medium. The resulting explants showed vigorous growth, with dark green leaves (Fig. 4a) and long roots (Fig. 4b).

**Table 8:** Variation analyses of rooting rate for different NAA and IBA combinations.

Variation source	*df*	MS	F	*P*-value
NAA	3	1558.764	83.091	< 0.001 **
IBA	1	3.190	0.170	0.686
Error	16	38.076		
Total	21			
* Represents a significant difference at *P* < 0.05. ** Represents a highly significant difference at *P* < 0.01. Abbreviations: *df* = degree of freedom; MS = mean square; NAA = naphthylacetic acid; IBA = indole butyric acid.				

### Factors influencing callus suspension culture

The best combination of conditions was determined with orthogonal tests, in which the proliferative effect of the suspension culture was obvious (Table 9). After 15 d of suspension culture, the average callus proliferation rate was 2–5 times that of initial callus quality (Table 9, Fig. 3e). After 30 d of suspension culture, all calli showed greater proliferative growth than that at 15 d, and the average callus proliferation rate was 3–11 times that of initial callus quality (Fig. 3f). Range analysis of suspension culture proliferation showed that the most important factor affecting callus proliferation at both X_1_ (15 d) and X_2_ (30 d) was B (inoculum), but for (X_2_–X_1_), the most important factor was A (medium type) (Table 10). The optimal medium for X_2_ and X_2_–X_1_ was MS medium with 4.5 g of calli and 120 mL of culture solution that was subcultured once without retaining the original culture solution (Table 10). ANOVA results showed that B (inoculum) had highly significant effects on X_1_ and X_2_, A (medium type) had highly significant effects on X_2_–X_1_, and D (retention of old medium) had significant effects on both X_1 _(15 d) and X_2_ (30 d) (Table 11).

**Table 9:** Results of orthogonal table for calli proliferation.

	X_I_ proliferation (g)	X_2_ proliferation (g)	(X_2_–X_1_) proliferation (g)	X_I_ proliferation coefficient	X_2_ proliferation coefficient	(X_2_–X_1_) proliferation coefficient
c1	7.51	17.97	10.46	5.00	11.98	6.97
c2	7.54	14.64	7.10	2.51	4.88	2.37
c3	8.69	15.93	7.25	1.93	3.54	1.61
c4	7.17	10.40	3.23	4.78	6.93	2.15
c5	10.08	12.33	2.25	3.36	4.11	0.75
c6	9.85	16.55	6.70	2.19	3.68	1.49
c7	6.54	11.37	4.83	4.36	7.58	3.22
c8	6.77	13.23	6.47	2.26	4.41	2.16
c9	9.09	19.80	10.71	2.02	4.40	2.38
X1 is the amount of calli proliferation after 15 d of suspension culture, X_2_ is the amount of calli proliferation after 30 d of suspension culture, (X_2_–X_1_) is the difference between the two calli proliferation rates. Proliferation coefficient = (proliferation amount – initial addition amount) / initial addition amount × 100.						

**Table 10:** Weight range analysis of suspension proliferation.

		A	B	C	D
X_1_	K1	23.74	21.21	26.68	−
	K2	27.10	24.38	23.93	−
	K3	22.40	27.64	22.62	−
	k1	7.91	7.07	8.89	−
	k2	9.03	8.13	7.98	−
	k3	7.47	9.21	7.54	−
	R	1.57	2.14	1.35	−
X_2_	*K1*	48.54	39.74	50.10	47.75
	*K2*	39.28	40.20	42.56	44.84
	*K3*	44.41	52.29	39.56	39.63
	*k1*	16.18	13.25	16.70	15.92
	*k2*	13.09	13.40	14.19	14.95
	*k3*	14.80	17.43	13.19	13.21
	*R*	3.09	4.18	3.51	2.71
(X_2_–X_1_)	*K1*	24.80	18.52	23.42	23.63
	*K2*	12.18	15.81	18.63	21.03
	*K3*	22.01	24.65	16.94	14.33
	*k1*	8.27	6.17	7.81	7.88
	*k2*	4.06	5.27	6.21	7.01
	*k3*	7.34	8.22	5.65	4.78
	*R*	4.21	2.95	2.16	3.10
Range = k(max) − k(mix); K_1_A = XA_1_ + XA_2_ + XA_3_, K_2_A = XA_4_ + XA_5_ + XA_6_, K_3_A = XA_7_ + XA_8_ + XA_9_…; kx = Kx/number of levels.					

**Table 11:** Variation analyses for suspension proliferation.

	Variation source	Sum of squares	*df*	MS	*F*	*P*-value
X_1_	Type of medium	9.36	2	4.68	0.917	0.413
	Addition of initial calli amount	100.107	2	50.053	37.797	0.000**
	Addition of initial liquid amount	7.167	2	3.583	0.69	0.511
X_2_	Type of medium	26.825	2	3.412	1.475	0.249
	Addition of initial calli amount	143.564	2	71.782	16.969	0.000**
	Addition of initial liquid amount	33.632	2	16.816	1.909	0.170
	Liquid retention	20.283	2	10.142	1.083	0.355
(X_2_-X_1_)	Type of medium	140.251	2	70.125	9.863	0.001**
	Addition of initial calli amount	62.952	2	31.476	3.047	0.066
	Addition of initial liquid amount	18.190	2	9.095	0.746	0.485
	Liquid retention	71.879	2	35.939	3.609	0.043*
*df*: degree of freedom; MS: mean square; Sig.: significance; * Represents a significant difference at *P* < 0.05. ** Represents a highly significant difference at *P* < 0.01.						

## DISCUSSION

Cytokinin is effective in promoting cell division and shoot regeneration and plays an important role in stem cell proliferation^[29,30]^. A commonly used cytokinin in tissue culture is 6-BA, which promotes shoot growth and leaf expansion and enhances chloroplast development^[31]^. Auxin also plays a role in callus formation^[32,33]^. When the concentration of auxin is higher than that of cytokinin, callus and root formation are promoted, and shoots are easily induced^[34,35]^. However, high cytokinin concentrations are often accompanied by vitrification and limited shoot development^[36]^. In this study, we found that high concentrations of 6-BA led to significant vitrification of explants. An earlier analysis of stem proliferation of *Malus* 'Yunxiangrong' showed that the number of clustered shoots first increased and then decreased with increasing 6-BA concentrations in *Prunus laurocerasus*^[37]^. When the 6-BA concentration reached a certain threshold, the number of shoots continued to increase, and stem shortening was observed^[38]^, which was consistent with our results (Fig. 2d).

The regeneration ability of *Sorbus* has been shown to be highly variable. For example, the regeneration rate of *S. alnifolia* leaves was 16%, but that of *S*. *folgneri* was 90%^[10,13]^. In this study, the regeneration efficiency of *S. caloneura* was similar to that of* S. alnifolia,* both of which were lower than 20%. In addition to species specificity, another reason for the low regeneration efficiency of adventitious shoots may be the long duration of the dark culture. Similar results were observed in the regeneration of adventitious shoots of poplar leaves, where adventitious shoots browned and died^[39]^. It has also been found that as culture time increases, the differentiation potential of organs and the regeneration ability gradually decrease, whereas browning increases^[40]^. These findings may therefore explain the low induction rate of adventitious shoots in our study, indicating that regeneration efficiency is closely related to explant genotype, hormone concentration and range, culture conditions, and other factors.

Plant cell suspension culture has emerged as a key technology for producing plant-specialized metabolites, which can promote rapid cellular and tissue proliferation, and the technology allows for a large number of uniformly dispersed and high-quality calli to be obtained easily^[41−43]^. Large-scale cell suspension culture is required to obtain a large number of calli in a short time to meet the demand for the production of secondary metabolites, which is important for industrial applications aimed at generating synthetic active ingredients^[44]^. In this study, the proliferation efficiency of the suspension system was high, with a more than 12-fold increase in the number of calli after 30 d. The establishment of a suspension system can potentially enable the industrial raising of seedlings and the production of a large number of secondary metabolites of *Sorbus*, such as biphenyl compounds, which can inhibit pathogenic bacteria and are widely used in the study of plant stress responses^[45]^. The growth of the plant suspension cell line followed an S-shaped curve, achieving slow, logarithmic, and stable phases^[46,47]^. The typical growth cycle of suspension cultures is approximately 1–2 weeks, and subculture is needed once every 5–7 d^[48]^. Zhuge & Que^[49]^ showed that the subculture cycle of *Cunninghamia lanecolata* suspension cells could not exceed 10 d. In a preliminary experiment, we found that the fresh weight of calli stabilized after approximately 15 d of culture, and Xiao et al.^[50]^ also reported similar results when they found that *S*. *aucuparia* suspension culture reached its maximum fresh cell weight at 14 d^[50]^. In this study, the subculture cycle was 15 d, and no browning was observed, indicating that the tissue quality did not decline. In addition, high initial inoculation density has been shown to be effective in establishing cell suspension cultures^[51]^. We found that the initial callus inoculation amount was the most important factor affecting callus proliferation and adding an appropriate amount of calli was beneficial to suspension culture proliferation. A small initial inoculation amount led to a long lag period due to the cell growth clustering effect. Moreover, suspended cells can only start to grow at a certain density, and their initial density is generally (0.5–2.5) × 10^5^ cells/mL^[52]^. In addition, when cells are densely populated in culture medium, the cell proliferation slows down or stagnates due to nutrient shortage. For *Pinus massoniana* cells, 1.0 g of callus in 30 mL of liquid medium was found to be the ideal condition for establishing a suspension system^[53,54]^. An inoculation amount of 30 g/L was most favourable for the establishment of the *Populus euphratica* suspension cell line^[55]^. Similarly, the optimal inoculation amount for a *Ginkgo* suspension cell line was 30–40 g/L^[56]^. Our results indicated that the best suspension culture conditions were 4.5 g of callus to 120 mL liquid medium, an inoculation amount of 37.5 g/L, and a ratio of callus-to-liquid per litre between 30 and 40, which is similar to the optimal inoculation concentration for suspension cultures of woody plants determined in other studies.

## CONCLUSIONS

To the best of our knowledge, this is the first report of stem proliferation, callus induction, rooting culture, and suspension culture of *S*. *caloneura*, as well as the first demonstration of adventitious shoot regeneration from leaves. We assessed the effects of multiple factors to identify the ideal conditions for culture and regeneration, providing a foundation for industrial seedling growth and genetic transformation of *S*. *caloneura*. Our findings also have implications for the breeding and genetic transformation of *Sorbus*.
